# Deciphering the *Nodamura virus* Protein A Function in *Schizosaccharomyces pombe* and Engineering a Novel Self-Amplifying RNA (saRNA) Vector NovaVec for Vaccine Development

**DOI:** 10.3390/vaccines14060532

**Published:** 2026-06-15

**Authors:** Xueyao Song, Ruihan Liu, Zhuo Zhang, Yuying Pan, Wanting Qu, Niubing Zhang, Xuan Li, Xiangping Yao, Pei Hao

**Affiliations:** 1 Key Laboratory of Synthetic Biology, State Key Laboratory of Plant Trait Design, CAS Center for Excellence in Molecular Plant Sciences, Chinese Academy of Sciences, Shanghai 200032, China; 2 University of Chinese Academy of Sciences, Beijing 100039, China; 3 Shanghai Institute of Materia Medica, Chinese Academy of Sciences, Shanghai 201203, China; 4 Department of Neurology, Fujian Institute of Neurology, The First Affiliated Hospital, Fujian Medical University, Fuzhou 350005, China

**Keywords:** self-amplifying RNA (saRNA), *Nodamura virus*, viral replication complex, RNA vaccine, RNA-dependent RNA polymerase

## Abstract

**Background/Objectives:** Self-amplifying RNA (saRNA) vectors enable high-level transgene expression from minimal initial doses. While alphavirus-based saRNA systems are widely used, they suffer from limitations, including large genome size, complex replicase machinery, and cellular toxicity. Nodamura virus (NoV) offers a promising alternative due to its compact genome (3.2 kb) and low cytotoxicity. This study aimed to elucidate NoV RNA1 replication mechanisms and develop a novel NoV-based saRNA vector platform. **Methods:** We established a *Schizosaccharomyces pombe* system to investigate NoV RNA1 replication and protein A localization. N-terminal deletion mutants and ER-targeting chimeras were constructed to characterize membrane targeting determinants. Based on mechanistic insights, we developed NovaVec by inserting transgenes at the RNA3^422^ site within the subgenomic RNA3 region. In vivo performance was evaluated using lipid nanoparticle-encapsulated NovaVec expressing nanoluciferase or monkeypox A33R antigen in BALB/c mice. **Results:** We identified redundant mitochondrial targeting domains (amino acids 2-15 and 16-33) in NoV protein A, where either domain was sufficient for proper localization and replication. The replication machinery could be functionally redirected to the endoplasmic reticulum while maintaining replication competence. Lipid nanoparticle-encapsulated NovaVec achieved sustained transgene expression for 54 days in mice, significantly outperforming conventional mRNA vectors that lost signal within 14 days. The NovaVec-based monkeypox A33R vaccine elicited robust antigen-specific humoral immunity with titers reaching approximately 1:12,800 following booster immunization. **Conclusions:** With its compact genome encoding only a single replicase protein, minimal cytopathic effects, and demonstrated capacity for long-term protein expression, NovaVec represents a highly promising next-generation saRNA platform for vaccines.

## 1. Introduction

Self-amplifying RNA (saRNA) vectors have emerged as a transformative platform for heterologous protein expression, with wide-ranging applications, like the development of next-generation vaccines [1,2]. By mimicking the self-amplifying machinery of positive-sense single-stranded RNA [(+)ssRNA] viruses, these vectors enable high-level transgene expression from minimal initial doses [3,4]. To date, the most extensively characterized saRNA systems are derived from alphaviruses, such as *Sindbis virus* (SINV), *Semliki Forest virus* (SFV), and *Venezuelan equine encephalomyelitis virus* (VEEV) [5,6,7,8]. However, the large genomic size of alphavirus replicons often presents significant challenges for heterologous gene size, delivery efficiency, and applicability in broad host environments for therapeutic purposes [4,9,10]. Consequently, there is a burgeoning interest in identifying alternative viral scaffolds with more compact genomes.

Nodamura virus (NoV), the type species of the genus Alphanodavirus (family Nodaviridae), offers a promising candidate for saRNA vector development. The NoV genome is bipartite, comprising two capped, non-polyadenylated RNAs: RNA1 (3.2 kb) and RNA2 (1.3 kb). RNA1 encodes protein A, a 112 kDa RNA-dependent RNA polymerase (RdRp) that is the sole viral factor required for the replication of both genomic segments [11,12]. During replication, a subgenomic RNA3 (sgRNA3, 0.5 kb) is synthesized from the RNA1 template, encoding proteins B1 and B2, the latter of which serves as a potent suppressor of RNA interference (RNAi) [13,14,15,16,17]. RNA2 directs the synthesis of a 43 kDa precursor to the viral coat proteins and replicates with the assistance of protein A. Critically, NoV RNA1 functions as an autonomous replicon, capable of initiating replication in a remarkably diverse array of hosts, ranging from insects to mammals [18]. Unlike the related Flock House virus (FHV), which is restricted to lower temperatures, NoV protein A exhibits robust thermal stability, supporting optimal replication at 28–31 °C and sufficient activity at 37 °C [18]. Furthermore, NoV infection is characterized by exceptionally high yields and minimal cytopathic effects in mammalian cells, such as BHK21, where cells remain viable for extended periods despite active viral replication [19,20,21]. The compact genome size, thermal stability, and low cytotoxicity position NoV as an attractive saRNA vector alternative to the VEEV-based systems [22]. Recently, the NoV replicon has been harnessed for saRNA vectors [23,24] and COVID-19 vaccine development [25], both of which were packaged in virus-like particles (VLPs). However, research on NoV replication mechanisms and vector development remains very limited when compared to that of alphaviruses. Exploring alternative vector designs based on the NoV replicon structure could facilitate the development of more efficient self-amplifying RNA vectors.

A fundamental requirement for the replication of (+)ssRNA viruses is the recruitment of host intracellular membranes to facilitate the assembly of viral replication complexes (RCs) [26,27,28]. These membrane-bound compartments serve to concentrate replicative components, sequester double-stranded RNA (dsRNA) intermediates from host innate immune sensors or assist in the proper folding of functional proteins [29,30,31]. Many (+)ssRNA viruses utilize the membranes of the endoplasmic reticulum (ER) or Golgi apparatus, while NoV specifically targets the outer mitochondrial membrane [21,32]. The localization of protein A is critical for RCs’ function. There are two membrane-associated regions (MARs) within NoV protein A crucial for its mitochondrial membrane localization and genome replication [21]. Further research is needed to elucidate the specific mechanism of protein A membrane binding, including the specific molecular determinants and selectivity for the organelle membrane. These detailed studies will help us understand the replication mechanism of NoV replicon that would assist the development of more efficient NoV-based vectors.

NoV is the only nodavirus known to infect mammals and lethally target suckling mice [18], exhibiting host conservatism in genome replication. Previous studies have established that NoV can replicate its entire genome in *Saccharomyces cerevisiae*, with RNA1 reaching levels exceeding 10^4^ copies per cell [33]. Notably, in that study, reporter genes were inserted into RNA2, necessitating co-expression of both RNA1 and RNA2 genomic segments for replications. To facilitate a simpler design and more compact saRNA vector, in this study we inserted reporter genes at the designated site of RNA1, enabling autonomous screening of RNA1-encoded functions without involving RNA2. The yeast *Schizosaccharomyces pombe* represents a powerful model system for dissecting the mechanisms of viral replication, offering a genetic environment that more closely mirrors higher eukaryotes than *S. cerevisiae* [34,35,36]. We established autonomous NoV RNA1 replication in *S. pombe* as a host, which allows for the visualization of protein A localization and the investigation of how differential subcellular anchoring influences replicative fitness. Using this system, we characterized the N-terminal determinants of NoV protein A for mitochondrial targeting. We also demonstrated that the NoV replication machinery possesses sufficient plasticity to be functionally redirected to the ER. Furthermore, based on the detailed dissection of NoV protein A function, we engineered the most compact NoV-based self-amplifying RNA vector, NovaVec, with a novel design. By inserting a gene of interest (GOI) into the 422nd base of the subgenome RNA3 (sgRNA3) sequence, which is located on the 3′-portion of RNA1, we achieved high-level transgene expression of GOI. An in vivo study in a mice model demonstrated that the NovaVec RNA delivered via lipid nanoparticles (LNPs) achieved sustained transgene expression for up to 54 days in mice. A NovaVec-based saRNA vaccine candidate against monkeypox A33R protein elicited robust humoral immunity, highlighting its promise for further preclinical development and for becoming effective immune therapeutics.

## 2. Materials and Methods

### 2.1. Materials and General Molecular Biology Techniques

Standard chemicals were supplied by Sigma-Aldrich (Shanghai, China) unless indicated otherwise. For routine molecular cloning and plasmid propagation, chemically competent *Escherichia coli DH5α* cells (Takara Bio Technology Co. Ltd., Beijing, China) were utilized. Target DNA amplification was executed using either KOD-FX DNA polymerase (TOYOBO, Osaka, Japan) or the HieffTM PCR Master MixTaq (Yeasen, Shanghai, China). The resulting PCR amplicons were resolved by agarose gel electrophoresis and subsequently recovered with an OMEGA Gel Extraction Kit (Omega Bio-tek, Norcross, GA, USA). To assemble recombinant vectors, we employed either the ClonExpress^®^ II One Step Cloning Kit (Vazyme, Nanjing, China) or standard restriction–ligation procedures using endonucleases and T4 DNA Ligase sourced from New England Biolabs (NEB, Ipswich, MA, USA) Following transformation, plasmid DNA was isolated via the OMEGA Plasmid Mini Kit I (Omega Bio-tek, Norcross, GA, USA). All constructed plasmids were ultimately validated through Sanger sequencing conducted by Tsingke Biotechnology (Shanghai, China).

### 2.2. S. pombe Strain, Transformation, and Culture Conditions

The *S. pombe* strain FY7652 (*h-leu1-32 ura4-D18*), obtained from the National BioResource Project (Osaka, Japan), served as the parental host for all genetic modifications. Routine cultivation was conducted at 30 °C in YES medium [37] (comprising 30 g/L glucose and 5 g/L yeast extract), which was appropriately supplemented with 50 μg/mL leucine and/or 50 μg/mL uracil. Liquid cultures were maintained under constant shaking at 220 rpm.

To introduce exogenous DNA, yeast cells were propagated to the mid-exponential growth phase and harvested for processing via the standard lithium acetate/PEG-mediated heat shock method [37]. Typical transformation reactions required either 100 ng of intact circular plasmids or 500 ng of linearized DNA. For chromosomal integration specifically, the pDuAL vectors were pre-digested with *Not*I in a 20 μL reaction volume to generate linear cassettes [38]. Following heat shock, the yeast cells were spread onto EMM minimal agar plates containing the requisite selective supplements (50 μg/mL leucine or 50 μg/mL uracil). Transformed colonies usually appeared on medium plates after incubation at 30 °C for 2–4 days.

### 2.3. Sequence Sources and Plasmid Constructions

The sequence of NoV RNA1 (accession: AF174533) was from the plasmid pMT-NoVRNA1 (kindly provided by Dr. Li’s lab, FDU) as described earlier in Yan Xu et al. 2021 [38] and was cloned into vectors pDuAL-HFF1 [39] and pCDNA3.1(+). For mRNA vaccine design, the sequence of MPV surface antigen A33R was obtained from the monkeypox virus reference sequence at NCBI (accession: GCF_014621545.1).

All plasmids were based on fission yeast multicopy and integration vector pDuAL-HFF1 and mammalian expression vector pCDNA 3.1, which contains the *URA4* and *LEU1* selectable marker gene. All primers (Supplementary Table S2) were synthesized by Genwiz Biotech (Suzhou, China). Plasmids and primer sequences in this study are listed in Supplementary Tables S2 and S3.

### 2.4. In Silico Predictions

We employed bioinformatics tools to predict the structure of the sequence by analyzing the membrane across the sequence. The sequence file was prepared using the FASTA format, and then analyzed online using the following tools: (1) HMMTOP [40] (www.enzim.hu/hmmtop/) was used with default parameter settings to predict the number of transmembrane helices in the target sequence, the location of the amino acids, and the transmembrane propensity (intra-/extracellular distribution); (2) TMHMM v.2 [41] (https://services.healthtech.dtu.dk/services/TMHMM-2.0/) was used with the hidden Markov model (HMM), setting the default probability limit and integrating the transmembrane propensity, electrical bias, peptide length, and transmembrane protein structure constraints (accessed on 5 March 2025). These tools predicted the transmembrane regions and the extracellular and intracellular regions, and the combined prediction results were used as the final transmembrane structure.

### 2.5. Fluorescence Imaging

Fluorescence imaging experiments were performed on a Leica TCS SP8 STED 3X (Leica Microsystems, Wetzlar, Germany) laser scanning confocal/super-resolution system. For yeast observations, a 100× oil immersion objective was used. The excitation and emission wavelengths for red fluorescence (mCherry) were 543 and 621 nm, and those for enhanced green fluorescent protein (eGFP) were 488 and 523 nm, respectively. Images were exported in TIFF format using Leica LAS X software LAS_X_4.7.0, preserving metadata information.

### 2.6. In Vitro mRNA Synthesis and Transfection Analysis

To prepare a template for mRNA synthesis, the NoVRNA1-RNA3^422^-Nluc DNA and NoVRNA1-RNA3^422^-A33R DNA cloning constructs were amplified by PCR using KOD DNA polymerase (TOYOBO, Osaka, Japan) with primers F: tactaatacgactcactataggtattgaatccaaaactcaaaatgctgaactacgagac; and R: cgagctctcccttagccatcc. Following linearization, the DNA templates were subjected to in vitro transcription utilizing the Vazyme EasyCap T7 Co-transcription Kit (Vazyme, Nanjing, China) equipped with the CAG Trimer. To isolate the synthesized transcripts, lithium chloride (LiCl) precipitation was initially performed. Subsequent elimination of residual DNA templates was achieved via DNase I digestion, and the final mRNA product was recovered utilizing an RNA Clean & Concentrator™ kit (Zymo Research, Irvine, CA, USA). For quality assurance, RNA concentrations were determined by NanoDrop spectrophotometry, while transcript purity and structural integrity were confirmed through standard agarose gel electrophoresis using the 0.5–10 kb ssRNA Ladder Marker of Takara.

### 2.7. mRNA@LNP Preparation and Characterization

The synthesis of mRNA-loaded lipid nanoparticles (LNPs) was performed by integrating four essential lipid components in a molar ratio consistent with the clinically validated Moderna formulation. Specifically, the lipid mixture comprised the ionizable cationic lipid SM−102, the helper phospholipid DOPE, structural cholesterol (Chol), and the pegylated lipid DMG−PEG2000, maintained at a precise molar percentage of 50%, 10%, 38.5%, and 1.5%, respectively. For the encapsulation process, the mRNA cargo was prepared in a 10 mM citrate buffer solution (pH 4.0), while the lipid constituents were dissolved in absolute ethanol to a final concentration of 10 mM. These two phases were introduced into a microfluidic device (Micro & Nano), where they were rapidly mixed at an aqueous-to-organic volumetric flow rate ratio of 3:1. The total flow rate was strictly controlled at 12 mL/min to ensure consistent particle formation, maintaining a target N:P ratio (defined as the ratio of nitrogen atoms in SM−102 to phosphate groups in mRNA) of 8:1.

Following the initial assembly, the raw LNP suspension underwent a rigorous purification and concentration procedure. The mixture was transferred to Millipore Amicon^®^ Ultra-15 centrifugal filter units with a 100 kDa molecular weight cut-off (MWCO). To facilitate efficient buffer exchange and remove residual ethanol, the units were centrifuged at 4000× *g* for a duration of 10 min at a controlled temperature of 4 °C. This process was repeated through two subsequent washing cycles using phosphate-buffered saline (PBS, pH 7.4) as the exchange medium. The final concentrated LNPs were passed through a 0.22 µm polyethersulfone sterile filter to maintain aseptic conditions and stored at 4 °C for subsequent analysis. The hydrodynamic diameter and polydispersity index (PDI) of the particles were characterized via dynamic light scattering (DLS) using a Zetasizer Nano ZS system. Furthermore, the encapsulation efficiency (EE) was determined utilizing a Quant-it™ Ribogreen assay. In brief, the LNPs were lysed using 1% Triton X-100 to liberate the encapsulated mRNA transcripts. The resulting fluorescence signal was measured and quantified by benchmarking against a standard curve of known mRNA concentrations. The final EE value was derived by calculating the percentage of sequestered mRNA relative to the total mRNA content within the formulation.

### 2.8. Animals, Biosafety, and Ethics

In this study, female BALB/c mice, aged 6 weeks, were acquired from VitalRiver Laboratory Animal Technology Co., Ltd. (Jiaxing, China) to serve as the experimental model. All procedures involving the use of live animals were conducted in strict accordance with institutional guidelines and received formal authorization from the Animal Welfare and Ethics Committee of the Shanghai Institute of Immunity and Infection (Approval No. A2023028). Prior to the commencement of experiments, the mice were housed in a high-standard specific pathogen-free (SPF) facility to ensure a controlled and healthy environment. The housing conditions were meticulously regulated, with the ambient temperature maintained within a stable range of 22 °C to 26 °C. The facility operated on a precise 12 h light/12 h dark photoperiod to support the natural circadian rhythms of the mice. Furthermore, all animal subjects were granted ad libitum access to a standardized nutritional diet and sterilized drinking water throughout the entire duration of the research period.

### 2.9. Bioluminescence In Vivo Imaging for Protein Expression Monitoring

Dynamic protein expression monitoring was achieved using the nanoluciferase-furimazine bioluminescence system integrated with the IVIS Spectrum spectral in vivo imaging system.

First, gene delivery was performed via intramuscular injection in BALB/c mice (*n* = 5, 6 weeks old). Each hind leg received an injection of LNP formulation containing 2 μg LNP-NovaVec-Nluc. Subsequently, the animals were anesthetized with inhaled isoflurane (induction concentration 5%, maintenance concentration 1.5–2%) and received an intraperitoneal injection of the substrate furimazine at 1.25 mg/kg body weight. The injection site was selected in the lower left abdomen to minimize visceral irritation. Imaging was performed on an IVIS Spectrum system equipped with a −90 °C cooled CCD camera and 28 narrow-band filters (430–850 nm). The imaging platform was preheated to 37 °C and maintained at a constant temperature prior to imaging. Based on the signal peak time determined in preliminary experiments (peaking 5–15 min post-injection), bioluminescence signals were acquired using DLIT (Diffuse Light Imaging Tomography) mode with exposure times ranging from 1 to 60 s (adjusted based on signal intensity). The 460 nm dedicated channel filter was selected. Following imaging, mice were monitored until complete recovery from anesthesia before being returned to their cages with free access to food and water.

### 2.10. Immunization and Serum Collection

For the evaluation of humoral immune responses, female BALB/c mice (6 weeks old, female, *n* = 6) were subjected to a standardized prime-boost vaccination protocol. The immunization regimen consisted of two separate intramuscular injections, each delivering a dose of 5 μg of the formulated NovaVec−A33R mRNA. The primary immunization (prime) was administered on day 0, followed by a secondary administration (booster) on day 14 to maximize the induction of specific antibodies. To monitor the kinetics of the immune response, peripheral blood was systematically collected at multiple time points, specifically on days 0, 7, 14, and 28 following the initial dose. The blood was harvested from the submandibular (cheek) vein utilizing a sterile lancet.

Following collection, the whole blood was incubated undisturbed at room temperature for a period of 30 min to facilitate the formation of a stable clot. The samples were then clarified by centrifugation at 2000× *g* for 10 min at a controlled temperature of 4 °C to effectively separate the serum from the cellular components. To eliminate the potential interference of endogenous complement activity in subsequent immunological assays, the isolated serum was subjected to heat inactivation in a water bath at 65 °C for an interval of 30 min. Finally, the processed serum was carefully partitioned into small aliquots to avoid repeated freeze–thaw cycles and stored at −80 °C for long-term preservation prior to downstream analysis.

### 2.11. MPV-Antigen A33R Expression in E. coli and Purification

To ensure the efficient soluble expression of the A33R antigen in a prokaryotic system, the genetic sequence of the protein was strategically modified. Specifically, the segments encoding the hydrophobic N-terminal signal peptides and the C-terminal transmembrane domains were accurately identified and excised through targeted PCR amplification. The resulting truncated A33R gene fragment was subsequently integrated into the pGEX−4T−1 expression vector. This molecular assembly was achieved using the infusion cloning technology, adhering to the protocols established in our previous research (Zhang et al., 2023) [42]. The recombinant constructs were then transformed into chemically competent Rosetta (DE3) *E. coli* cells (obtained from WEIDI, Shanghai, China), a strain chosen for its ability to enhance the expression of eukaryotic proteins by providing rare tRNAs.

The protein expression was initiated by cultivating the transformed cells until they reached the logarithmic growth phase. At this point, the synthesis of the recombinant MPV-antigens was stimulated by the addition of isopropyl-β-D-thiogalactoside (IPTG, Beyotime) at a final concentration of 300 μmol/L. To facilitate proper protein folding and mitigate the formation of insoluble inclusion bodies, the induction process was conducted at a lowered temperature of 16 °C for an extended duration of 18 h. Following the induction period, the bacterial cells were harvested and lysed. The target recombinant proteins were then isolated and refined through affinity chromatography. Depending on the specific fusion tag utilized, either Ni−NTA agarose resin (Yeasen) or GST-tag purification resin (Beyotime) was employed, following the detailed operational guidelines provided by the respective manufacturers to ensure high purity for subsequent assays.

### 2.12. ELISA Coating and Antibody Detection

We used purified recombinant proteins to determine the IgG antibody titer against the MPV A33R antigen by ELISA. We coated 5 μg/mL of recombinant protein on a 96-well plate (Corning, NY, USA) and incubated it at 4 °C overnight. We washed the plate with TBST (20 mM Tris, 137 mM NaCl, 0.1% Tween, pH 7.4) and blocked it with 5% skim milk at 37 °C for 1 h. Then, we washed the plate three times with TBST and added a series of dilutions of heat-inactivated mouse serum. We incubated the plate at 37 °C for 1 h, washed it three times with TBST, and added HRP-conjugated goat anti-mouse IgG (Yeasen, diluted 1:5000 with 1% skim milk/TBST) at 37 °C for 1 h. Then, wash three times with TBST. After the final wash, incubate with 3,3′,5,5′-tetramethylbenzidine (MesGen, Shanghai, China) for 15 min, and then terminate the reaction with 2 mol L^−1^ HCl. Record the absorbance at 450 nm using a Varioskan Flash microplate reader (Thermo Fisher Scientific, Waltham, MA, USA).

## 3. Results

### 3.1. Exploring NoV RNA1 Replication in S. pombe

**Figure 1:**
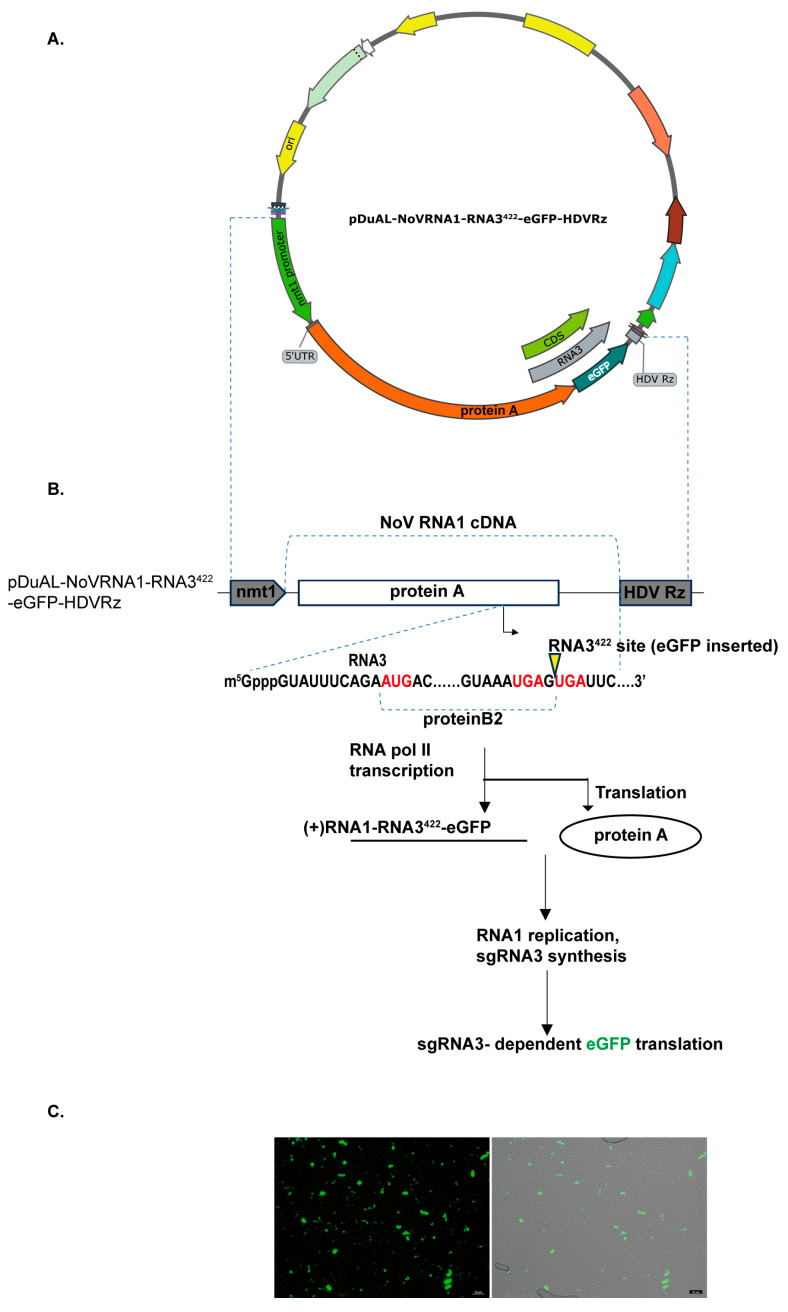
NoV RNA1 replication-dependent eGFP expression in *S. pombe*. (**A**) Plasmid map of pDuAL-NoVRNA1-RNA3^422^-eGFP-HDVRz. (**B**) Working pathway for pDuAL-NoVRNA1-RNA3^422^-eGFP-HDVRz to express replication-dependent eGFP in *S. pombe*. NoV RNA1 cDNA was inserted downstream of the *nmt1* promoter in the yeast vector pDuAL-HFF1. Bases marked in red font are the start codon of protein B2, the stop codon of protein A and the stop codon of protein B2 from 5′ to 3′, respectively; the yellow triangle points to the site where eGFP is inserted (called the RNA3^422^ site). pDuAL-NoVRNA1-RNA3^422^-eGFP-HDVRz undergoes RNA pol II transcription and translation to produce positive-sense the NoV genome RNA1, which has eGFP inserted at RNA3^422^ [(+) RNA1-RNA3^422^-eGFP] and protein A. Protein A replicates (+) RNA1-RNA3^422^-eGFP and synthesizes sgRNA3, using (+) RNA1-RNA3^422^-eGFP as a template. (**C**) Fluorescent images demonstrate eGFP expression in yeast colony positive for the transfected pDuAL-NoVRNA1-RNA3^422^-eGFP-HDVRz. Scale bar = 25 μm.

Given the host conservatism of the NoV genome replication, we established an *S. pombe* system of NoV genome replication to facilitate investigation of NoV RNA1. To enable stable genetic manipulation, the cDNA of NoV RNA1 was cloned into the yeast expression plasmid pDuAL-HFF1 (Figure 1A). In principle, the design of a reporter gene embedded within sgRNA3 would produce high copy numbers of mRNA strictly dependent on NoV-specific negative-strand synthesis and subsequent subgenomic transcription, which can produce abundant reporter protein. We selected enhanced green fluorescent protein (eGFP) as the reporter to allow for monitoring in living cells. However, insertion strategies were constrained by the compact genomic architecture of RNA1, particularly the extensive overlap between the 3′ end of the protein A open reading frame (ORF), the sgRNA3 sequence, and the protein B2 ORF [43]. To avoid disrupting protein A, the only factor required for NoV genomic replication [32], we inserted the eGFP ORF at the 422nd base of the sgRNA3 sequence, which was between the protein A termination codon and the protein B2 stop codon (Figure 1B). This site was named RNA3^422^, which preserved protein A integrity while generating a protein B2-eGFP fusion.

In this design, (+) RNA1 transcription was driven by the *nmt1* promoter within the plasmid pDuAL-NoVRNA1-RNA3^422^-eGFP-HDVRz. The self-cleaving hepatitis delta virus ribozyme (HDV Rz) was positioned at the 3′ end to ensure the generation of an authentic viral 3′ UTR. Upon translation from the primary (+) RNA1 transcript, protein A uses the (+) RNA1 transcript as a template to synthesize progeny (−) RNA1, from which both (+) RNA1 and sgRNA3 were generated. Then the protein B2-eGFP fusion was translated from sgRNA3 (Figure 1B), in turn producing green fluorescence. So green fluorescence-producing cells represented active replication of NoV RNA1.

Fluorescence microscopy of living yeast cells containing the pDuAL-NoVRNA1-RNA3^422^-eGFP-HDVRz plasmid revealed green fluorescence (Figure 1C). This confirms that protein A mediates functional replication of the primary DNA-derived RNA1 transcript, resulting in the successful synthesis of sgRNA3, signifying the successful establishment of the *S. pombe* system for NoV genome replication. Notably, we observed a clear distinction between fluorescent and non-fluorescent cell populations, a phenomenon also reported in the study of FHV, which was suggested to be attributed to incomplete plasmid segregation in yeast cells [44].

### 3.2. Analyzing Determinants for NoV Protein A Mitochondrial Localization and Replication Function in S. pombe

**Figure 2:**
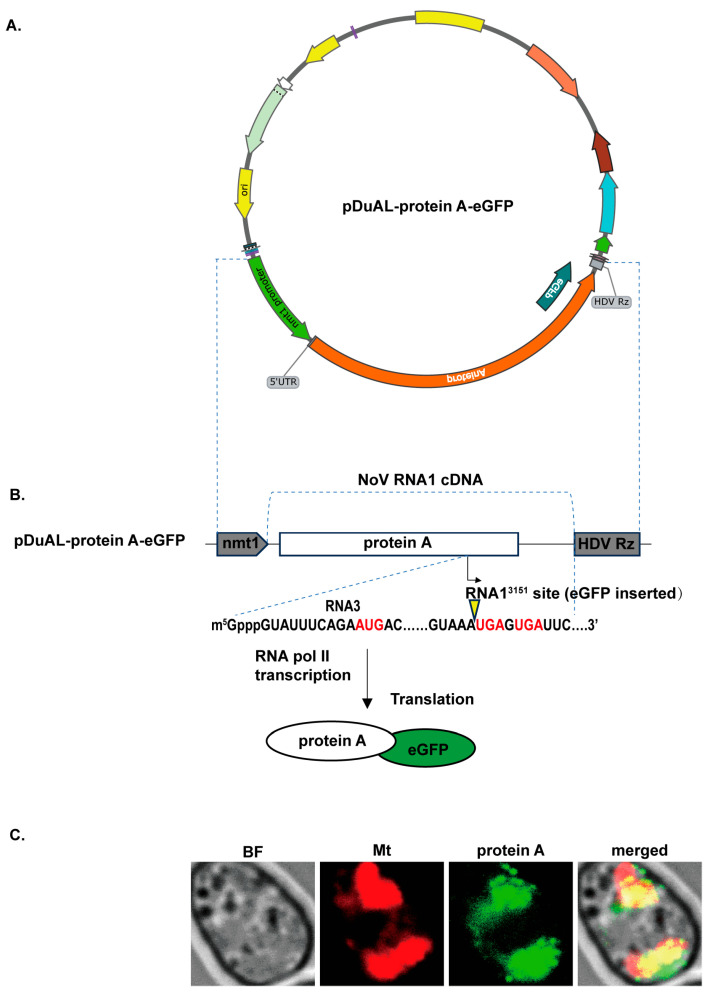
Protein A colocalizes with mitochondria in *S. pombe*. (**A**) Plasmid map of pDuAL-protein A-eGFP. (**B**) Working pathway for pDuAL-protein A-eGFP. Bases marked in red font are the start codon of protein B2, the stop codon of protein A and the stop codon of protein B2 from 5′ to 3′, respectively; the yellow triangle points to where the site eGFP is inserted (called the C1 site). (**C**) Fluorescent confocal images of yeast cells. BF: bright field of yeast cells; Mt: red fluorescently labeled mitochondria; protein A: green fluorescently labeled protein A; merged: red fluorescence and green fluorescence overlay the field.

The localization of RNA replicons to intracellular membranes is a common feature of (+)ssRNA viruses and is critical for the assembly and function of viral RCs. To visualize the subcellular localization of NoV protein A in *S. pombe*, we generated a construct, pDuAL-protein A-eGFP, by inserting the eGFP ORF at the 3151st base of the RNA1 sequence, which was immediately upstream of the protein A termination codon, leading to the expression of a protein A-eGFP fusion protein. This site was named RNA1^3151^ (Figure 2A,B).

NoV protein A is known to localize to the outer mitochondrial membrane in BHK21 cells [21]. To determine whether this localization was conserved in a heterologous host, we generated an *S. pombe* strain with mitochondria labeled with red fluorescence, which was designated as Red-Mito. The red fluorescence was generated by mCherry fused to the outer mitochondrial membrane-targeting sequence of Tom70 (Figure S1). In Red-Mito yeast transformed with pDuAL-protein A-eGFP, we observed co-localization of protein A in the mitochondria [Pearson correlation coefficient (PCC) = 0.89; Table S1, Figure 2C], indicating a high degree of spatial correlation.

**Figure 3:**
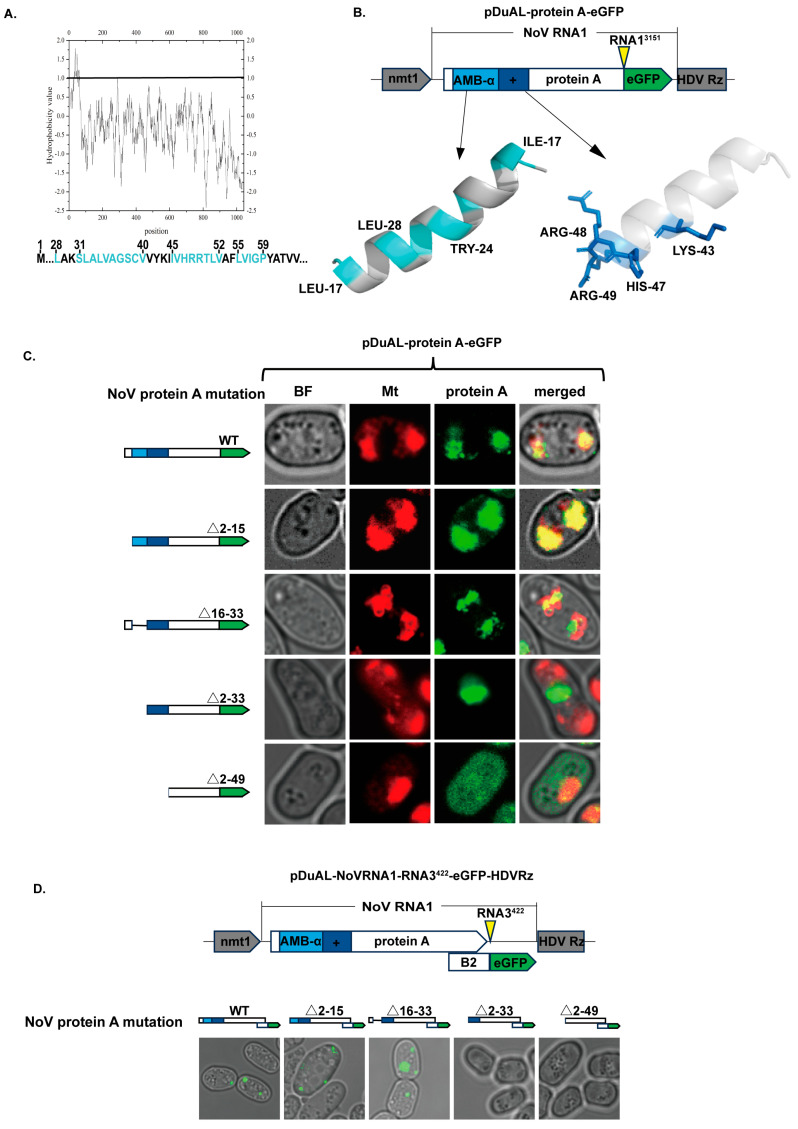
N-terminal sequence of protein A affects its subcellular localization and function. (**A**) Hydrophobicity prediction results for the full amino acid sequence of protein A and the show of amino acids 1-64 of protein A. Black: amino acids (hydrophobicity value < 1); cyan: amino acids (hydrophobicity value > 1). An amino acid that has a hydrophobicity value greater than 1 (up from the solid line) is considered to indicate great hydrophobicity. (**B**) Schematic diagram of the N-terminal predicted amphipathic α-helix and positively charged amino acid region of protein A. Cyan-labeled amino acids: hydrophobic amino acids; blue-labeled amino acids: positively charged amino acids. (**C**) Four structural variants of the N-terminal region of protein A are presented. Δ2-15: protein A mutant with amino acids 2-15 deleted; Δ16-33: protein A mutant with amino acids 16-33 deleted; Δ2-33: protein A mutant with amino acids 2-33 deleted; Δ2-49: protein A mutant with amino acids 2-49 deleted. Representative confocal images for NoV protein A (third column, green), mitochondria (second column, red), merged signals (fourth column) and positive yeast clones of pDuAL-protein A-eGFP with mutations at the corresponding positions are shown. The merged images represent a digital superimposition of red and green signals, where areas of fluorescence colocalization are yellow. (**D**) Representative fluorescent photographs of positive yeast clones of pDuAL-NoVRNA1-RNA3^422^-eGFP-HDVRz with mutations at the corresponding positions are shown.

Deletion of amino acids 12-64 of NoV protein A in a previous study confirmed the region’s function for mitochondrial targeting, which contained two predicted MARs (amino acids 12-34 and 42-64) [21]. Within these MARs, a predicted amphipathic α-helix (amino acids 16-33) was suggested to play a critical role in membrane targeting [45]. Our analyses identified a hydrophobic signal near the N-terminus of NoV protein A (amino acids 28-59) (Figure 3A). Although this region is enriched in hydrophobic residues, topology predictions indicated that it does not form a conventional transmembrane domain (TMD) (Figure S2B). Also in the N-terminal region of protein A, we found a positively charged region containing several positively charged amino acids, amino acids 43, 47, 48 and 49, that may facilitate interactions with negatively charged membrane surfaces (Figure 3B).

To investigate the roles of these N-terminal domains in membrane association and replication function of protein A, we generated a series of N-terminal deletion mutants of protein A: Δ2-15 (region in front of the amphipathic α-helix), Δ16-33 (region of the amphipathic α-helix), Δ2-33 and Δ2-49 (targeting region containing positively charged amino acids) (Figure 3C, left panel). An eGFP reporter was inserted at the RNA1^3151^ site for producing the protein A-eGFP fusion protein (Figure 3B). These constructs were transformed into the Red-Mito strain. Subcellular localization and replication capacity were determined via confocal microscopy, for which co-localization with mitochondria was quantified using PCC values (Figure 3C, right panel, and Table S1).

Confocal microscopy revealed distinct localization patterns among the mutants. The deletion of amino acids 2-15 (Δ2-15) did not alter mitochondrial localization relative to the wild-type protein A, indicating that this region was dispensable for targeting, while deletion of the predicted amphipathic α-helix (Δ16-33) slightly reduced mitochondrial co-localization (PCC = 0.49), although a peri-mitochondrial distribution persisted. However, the deletion of both the upstream and the helical regions, Δ2-33, resulted in loss of mitochondrial localization and complete dislocation of the mutant protein A. These results indicated protein A’s N-terminal region contained at least two functional domains, in which sequence 2-15 or 16-33 had the redundant function of targeting protein A to the mitochondrial membrane, and one of them was sufficient for the correct localization of protein A. Further extension of the deletion to include the downstream positively charged region (containing amino acids 43, 47, 48 and 49), Δ2-49, produced a diffusely distributed protein with no apparent organelle association, suggesting membrane-targeting function of the positively charged region. And these regions act synergistically to anchor protein A to mitochondrial membranes. Our findings provided fine mapping of N-terminal functional domains of protein A.

To investigate whether these mutant forms of protein A support replication function, we inserted eGFP into these mutant constructs at the RNA3^422^ site, which was transformed into wild-type yeast. The expression of protein B2-eGFP fusion from sgRNA3 was mediated by replication of NoV RNA1. So green fluorescence-producing cells represented active replication of NoV RNA1. Green fluorescence was observed in wild-type protein A construct and mutant constructs, Δ2-15 and Δ16-33, but not in mutant constructs Δ2-33 and Δ2-49. These results suggested mitochondrial membrane association of protein A was strictly required for NoV RNA1 replication, and dislocation of protein A completely abolished replication activities (Figure 3D).

### 3.3. Engineering Protein A for Retargeting to an Alternative Intracellular Membrane

**Figure 4:**
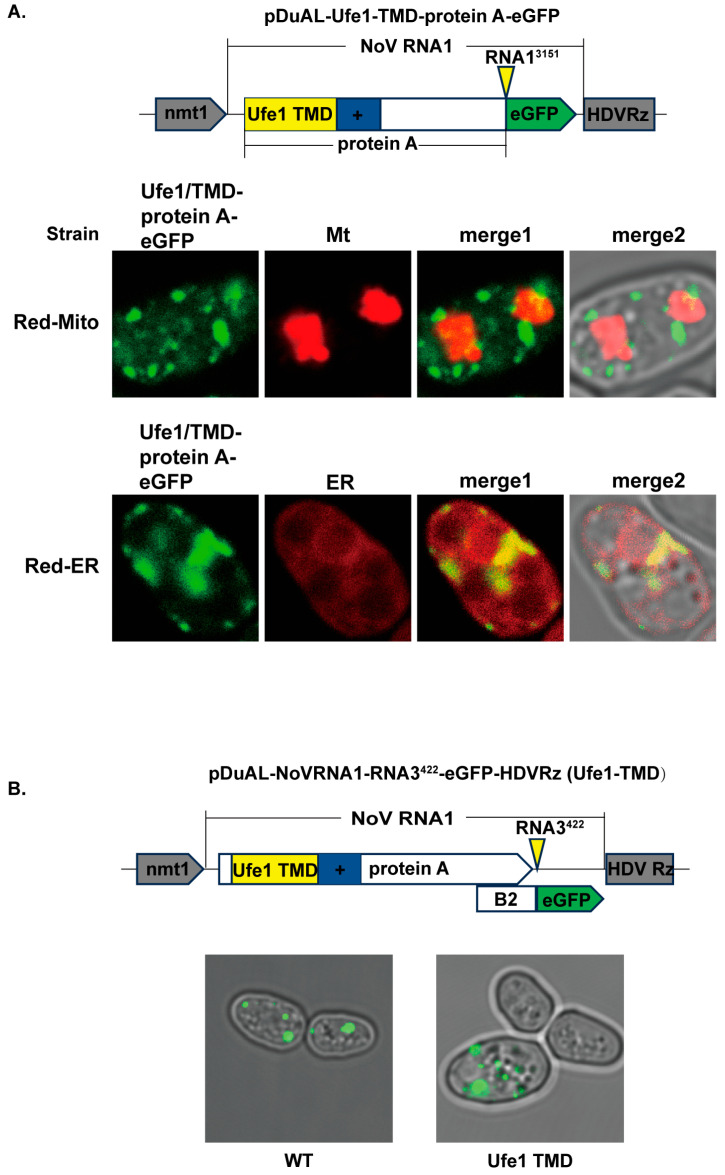
Chimeric protein A was retargeted to the ER and retained its activity in replication. (**A**) Schematic diagram of pDuAL-Ufe1-TMD-protein A-eGFP: amino acids 2-33 of protein A were replaced by ER-targeted Ufe1 TMD. Representative images for Red-Mito yeast transformed with pDuAL-Ufe1-TMD-protein A-eGFP (first line) and Red-ER yeast transformed with pDuAL-Ufe1-TMD-protein A-eGFP (second line). (**B**) Representative fluorescent photographs of positive yeast clones of wild-type protein A construct (first image) and the Ufe1/TMD-protein A-eGFP fusion construct (second image) are shown.

By replacing the native mitochondrial targeting signal with an ER-targeting sequence from yeast *S. cerevisiae*, it was previously shown that alternative intracellular membranes can support FHV replication [32]. To test whether retargeting NoV protein A to the ER could restore its replication activity, we replaced its mitochondrial targeting region (amino acids 2-33) with a heterologous ER-targeting sequence, for which the inverted C-terminal TMD of the yeast t-SNARE Ufe1p (amino acids 326-346) was used [32,46] (Figure 4A). To visualize localization in this context, we generated an *S. pombe* strain designated Red-ER, with ER labeled with red fluorescence using mCherry fused to the inverted C-terminus of Ufe1p (Figure S3). A chimeric protein A construct was made by fusing the Ufe1 TMD, protein A and eGFP (Figure 4A top panel). This construct was transformed into the Red-ER strain and into the Red-Mito strain as a control. In Red-ER, the Ufe1/TMD-protein A-eGFP fusion protein that emitted green fluorescence was found to be co-localized with mCherry-labeled ER (red fluorescence), suggesting the chimeric protein A was successfully redirected to the ER in the Red-ER strain (Figure 4A bottom panel). Conversely, control experiments performed in Red-Mito yeast showed separation of chimeric protein A from mitochondria, validating losing mitochondria-targeting signal and dislocation from mitochondria. These results demonstrated that the substitution of the native N-terminal region with sequences from Ufe1p successfully redirected NoV protein A to the ER.

We subsequently assessed the functional consequences of this re-localization by monitoring RNA replication in vivo. To investigate whether the ER-targeted protein A supports the replication function, we inserted eGFP into the chimeric construct at the RNA3^422^ site (Figure 4B, top panel) and transformed it into the wild-type strain. Again, green fluorescence from the B2-eGFP fusion protein translated from sgRNA3 would represent active replication of NoV RNA1. Green fluorescence was observed in the wild-type protein A construct and the Ufe1/TMD-protein A-eGFP fusion construct (Figure 4B, bottom panel). These results suggested that the redirected protein A was properly structured and also functionally competent. Utilizing the *S. pombe* system for NoV genome replication, we showed for the first time that NoV protein A was organelle-flexible, capable of initiating replication from alternative intracellular membranes, and that membrane association was essential for RNA1 replication.

### 3.4. Constructing a Self-Amplifying RNA Vector NovaVec and Its In Vivo Applications in Vaccine Development

**Figure 5:**
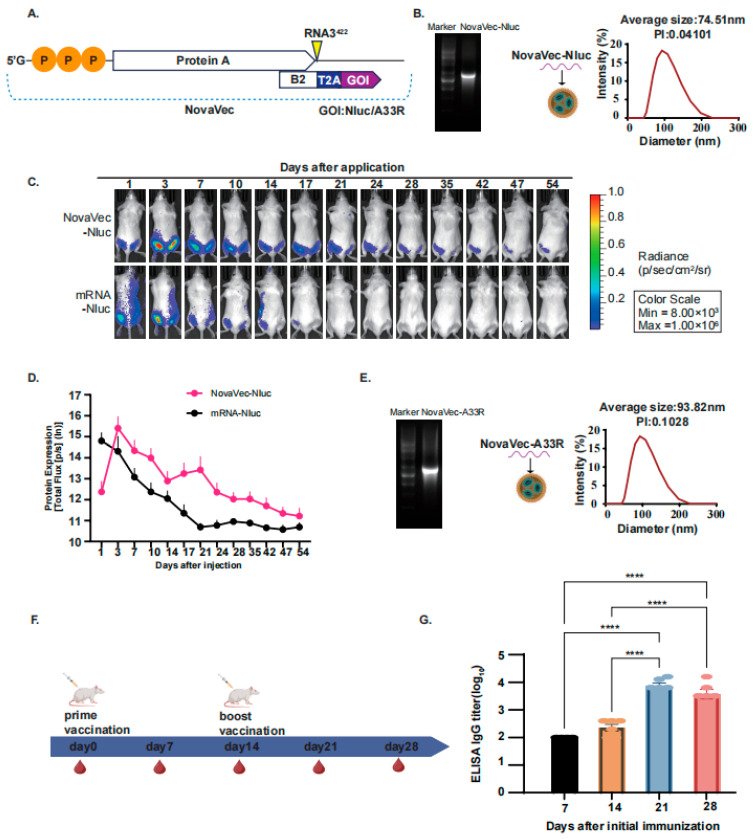
LNP-NovaVec system for live imaging and monkeypox saRNA vaccine in mice. (**A**) Schematic diagram of NovaVec RNA. (**B**) The agarose gel electrophoresis image of NovaVec-Nluc RNA and dynamic light scattering (DLS) data of LNPs that encapsulated NovaVec-Nluc RNA. (**C**) BALB/c mice were intramuscularly injected with LNP-NovaVec-Nluc or LNP-mRNA-Nluc (2 μg per hind leg, *n* = 5). At 1, 3, 7, 10, 14, 17, 21, 24, 28, 35, 42, 47, 54 days after injection, expression was visualized using an IVIS spectrum in vivo imaging system. (**D**) Quantitative statistics of total fluorescence values in live imaging. (**E**) The agarose gel electrophoresis image of NovaVec-A33R RNA and dynamic light scattering (DLS) data of LNPs that encapsulated NovaVec-A33R RNA. (**F**) Immunization and blood collection schedule scheme using BALB/c mice (5 μg per dose, *n* = 6). (**G**) Endpoint titers (log_10_) showed a ~2-fold increase in antibodies eliciting post-second immunization. Two-way ANOVA was used to compare between groups using pairwise multiple comparison, followed by Tukey’s multiple comparison test. **** indicates *p* < 0.0001.

Based on the analysis of the NoV RNA1 self-amplifying system in *S. pombe*, we engineered a novel NoV-based self-amplifying RNA vector, NovaVec, for short. NovaVec retained the NoV RNA1 sequence, with the RNA3^422^ site designated as the insertion locus for genes of interest (GOI), where the T2A self-cleaving peptide was used to separate GOI from protein B2 (Figure 5A). To test and validate NovaVec for in vivo transgene expression, we first tested the expression of the nanoluciferase (Nluc) in mice by inserting its ORF at the RNA3^422^ site to generate the construct NovaVec-Nluc (Figure 5A). In vitro transcribed NovaVec-Nluc RNA was identified by agarose gel electrophoresis, and the band was correctly observed in the 3000–4000 nt range. Then purified RNA was encapsulated in lipid nanoparticles (LNPs), which were assessed for size and uniformity (Polydispersity Index, PI) (Figure 5B). Intramuscular injection of LNP-encapsulated NovaVec-Nluc into BALB/c mice was performed, while LNP-encapsulated regular mRNA expressing Nluc was administered as a control. For NovaVec-Nluc, bioluminescence imaging detected signals that began 1day post-injection (dpi), peaked on 3 dpi, and persisted for at least 54 days (Figure 5C,D). For the Nluc mRNA as a control, the Nluc signals decayed quickly and lost signal 14 days post-injection. These data showed that NovaVec supports efficient, long-term foreign protein expression in animals, demonstrating its great potential for therapeutic applications.

We previously developed a multi-valent mRNA vaccine against monkeypox enveloped or mature virion surface antigens using linear mRNA and LNP [42]. To evaluate NovaVec as a vaccine platform, here we developed a monkeypox saRNA vaccine by inserting the mpox EV-antigen A33R into the NovaVec RNA3^422^ site to generate NovaVec-A33R. In vitro transcribed RNA was identified by agarose gel electrophoresis, and the band was correctly observed in the 3000–4000 nt range. Purified RNA was then encapsulated into LNPs and assessed for size and PI (Figure 5E). Immunogenicity was evaluated using a prime-boost strategy in BALB/c mice (Figure 5F). Animals received intramuscular injections of LNP-NovaVec-A33R on days 0 and 14, with PBS administered as a placebo control. Blood samples were collected via submandibular bleeding prior to immunization and on 7, 14, 21, and 28 days post-prime immunization (Figure 5F). Antigen-specific antibody responses were quantified by enzyme-linked immunosorbent assay (ELISA) utilizing recombinant A33R as the capture antigen (see Section 2). The LNP-NovaVec-A33R vaccine elicited robust humoral immune responses; antigen-specific IgG against A33R was detectable by day 7 post-prime immunization, indicating rapid immune activation. Antibody titers increased significantly following the secondary immunization, reaching titers of approximately 1:400 (prime) and 1:12,800 (boost) (Figure 5G). These findings demonstrated the utility of the NovaVec as an saRNA vaccine platform, highlighting the great potential of NovaVec for developing vaccines against emerging pathogens.

## 4. Discussion

This study demonstrates the bioengineering of NovaVec, a novel self-amplifying RNA (saRNA) platform derived from the NoV RNA1 replicon, and systematically elucidates the replicative mechanisms related to membrane targeting of its replicase protein A. By establishing a NoV replication system in *S. pombe*, we have revealed the molecular determinants of protein A mitochondrial localization and demonstrated the organelle-membrane plasticity of NoV replication machinery. These mechanistic studies guided the construction of the NovaVec saRNA platform, followed by the in vivo demonstration of its utilities in animals as an efficient saRNA platform. Our findings highlight the potential of NovaVec as a compact and efficient platform for vaccine development, addressing key limitations of current alphavirus-based saRNA systems.

The ability to study nodavirus replication in yeast, first established for FHV, provided a genetically tractable platform for dissecting viral mechanisms under strictly defined conditions. Price et al. demonstrated DNA-directed expression of functional FHV RNA1 derivatives in *S. cerevisiae* [44] and subsequently showed NoV replication in *S. cerevisiae* [33]. In this research of NoV, they used the genomic RNA2 to characterize the replication of the NoV genome, which required the simultaneous transformation of two independent genome segments that expressed NoV protein A and NoV RNA2 in a single *S. cerevisiae* cell. Differently in the current study, we used subgenome RNA3 to indicate the replication of NoV genomic RNA1 by inserting the reporter gene eGFP at the RNA3^422^ site of the RNA1 sequence. In addition, to characterize the subcellular localization of protein A, we engineered the protein A-eGFP fusion protein by inserting the reporter gene eGFP at the RNA1^3151^ site of the RNA1 sequence. Our design provided a direct readout of replication complex function using indication (green fluorescence) of sgRNA3 synthesis, greatly simplifying replication detection over the RNA2 system previously used [33]. Notably, protein A expression was evaluated via GFP fusion rather than Western blot, as this approach facilitates live-cell imaging of subcellular localization and replicase behavior, consistent with established methodologies for other positive-strand RNA viruses. The fluorescence intensities and patterns confirmed that all protein A variants were effectively expressed and functional (Figure 3C). In addition, we observed the fluorescence heterogeneity in positive yeast populations, which paralleled phenomena reported by Price et al. (2000) for FHV, which was attributed to incomplete plasmid segregation [44]. Based on this assumption, we engineered chromosomal integration of the NoV vector in the *S. pombe* genome by inserting the NoV RNA1 locus into the chromosome of *S. pombe*, ensuring that the genetic segment was evenly distributed across single yeast cells, providing a uniform system for precise mutational analysis [47] (Figure S4).

Using the reporter system of NoV protein A localization, we found that protein A localized to the mitochondria in *S. pombe*, which is consistent with its localization pattern in mammalian cells [21], confirming the functional conservation of protein A across host species. Gant et al. (2014) have predicted two MARs (amino acids 12-34 and 42-64) in protein A [21]. When they deleted amino acids 12-64 from protein A, it lost the ability to colocalize with mitochondria and lost replication activity [21]. In this study, we did further fine mapping of the N-terminal functional domain of NoV protein A. Our analysis suggested fine structure motifs that represent independent function units. Our results indicated that amino acid regions 2-15 and 16-33 can independently direct the mitochondrial targeting of protein A in *S. pombe*. The presence of either domain ensured the mitochondrial localization and replication activity of protein A, whereas the absence of both resulted in dislocation and loss of replication activity. We also identified a positively charged region containing amino acids 43 (Lys), 47 (His), 48 (Arg) and 49 (Arg) that can play a critical role in membrane targeting. Further deletion of the positively charged region resulted in a diffused distribution pattern with no apparent organelle association, suggesting roles of positively charged residues to facilitate interactions with negatively charged membrane surfaces. Taken together, the three domains, amino acids 2-15, 16-33, and 34-49, acted synergistically to anchor protein A to mitochondrial membranes. These complementary findings refined our understanding of protein A membrane-binding determinants, providing a more detailed characterization of the N-terminal regions required for protein A localization. The positive correlation between membrane binding and replication efficiency was consistent with findings from studies on FHV protein A and alphavirus replicases [31,48,49], suggesting that in *S. pombe*, which possessed a well-established small interfering RNA (siRNA) system [50], membrane association, as a prerequisite for the formation of RCs, could protect replication intermediates dsRNA and concentrate factors for replication. Our analysis of NoV protein A membrane targeting revealed distinct mechanisms compared to the related nodavirus, FHV. Miller and Ahlquist (2002) demonstrated that FHV protein A was a transmembrane protein with N-terminal sequences containing a 19-amino-acid TMD that was essential and sufficient for outer mitochondrial membrane targeting and membrane insertion [22]. Unlike FHV protein A, NoV protein A lacked a classical TMD and used an amphipathic α-helix to dock with the mitochondrial membrane without needing a penetrating TMD (Figure S2). Notably, within the *Nodaviridae* family, the structural divergence for membrane anchoring between FHV and NoV proteins suggested distinct strategies evolved in natural selection.

Although native NoV protein A uses a mechanism of membrane anchoring different from the transmembrane domain, we showed the TMD mechanism could function appropriately to replace NoV protein A’s native structure for membrane anchoring and support NoV genome replication. We replaced the native mitochondrial targeting sequence with an ER-targeting sequence from yeast Ufe1p that was a domain mediating transmembrane binding [32], which successfully retargeted protein A to the ER and retained the RNA replication activity of protein A. These results indicated NoV protein A is mechanism-flexible for membrane anchoring. Further, our study confirms, for the first time for NoV, that the replication machinery exhibits remarkable organelle flexibility, capable of initiating replication from alternative intracellular membranes. NoV RNA RCs retargeted from the mitochondria to the ER were functional, which was consistent with studies on FHV and *Tombusviruses* [32], confirming the organelle flexibility of viral replicases. The similar results suggested that interactions between viral replicase proteins and host intracellular membranes, but not the origin of the intracellular membranes, were necessary for positive-strand RNA virus RCs’ formation and function. Both ER and mitochondria supported comparable replication competence, suggesting that the fundamental requirement for viral RCs is the availability of a membranous surface rather than specific organelle functions. The ER was specifically chosen because it provides the most abundant membrane system and a large surface area, which may be beneficial for viral replication. Further retargeting studies with NoV, FHV, and other positive-strand RNA viruses and more attempts at relocating various organelles may identify broadly applicable principles. ER-retargeting studies of NoV were conducted in the yeast system due to its feasibility and the availability of tools for organelle manipulation. It is crucial to explore the performance of a hetero-organelle-located replicon in mammalian cells for future research. Specifically, the mitochondria-targeting region (aa 12-64) is functionally conserved between yeast and mammalian systems, suggesting that relocating protein A to the ER in mammalian cells can likely ensure proper assembly of the replication complex. Such membrane-based replication complexes serve to protect dsRNA intermediates and facilitate the enrichment of replication substrates. These findings in the yeast system support the translation to the mammalian system for the conservatism of the replication of the NoV replicon over various hosts [18,21].

The COVID-19 pandemic has accelerated the development of RNA-based vaccines, with saRNA vectors emerging as a promising alternative to conventional mRNA vaccines due to their ability to amplify antigen expression from minimal doses. The NovaVec platform offers significant advantages over existing alphavirus-based saRNA systems. Traditional alphavirus-based saRNA vectors (such as those developed from VEEV) made important progress in clinical trials [51,52,53,54]. However, their large genomes (∼9–15 kb) and replicase complex machinery (encoding four non-structural proteins) limit their applications due to the delivery challenges and potential cytotoxicity [9]. In contrast, NoV RNA1 is only 3.2 kb, encoding a single-protein replicase, protein A. Notably, NoV infection is characterized by high viral yields with only mild cytopathic effects, allowing host cells to remain viable for extended periods despite active viral replication [18]. This compact, minimalist design highlights the advantages of the NoV replicon for the development of saRNA vectors for vaccine development.

Recent years have seen growing interest in exploiting the NoV replicon for saRNA vectors. Recently, Karan et al. (2024) utilized plant virus-like particles (VLPs) to package the NoV RNA1-based replicon that fused the reporter gene eYFP to the C-terminus of protein A separated with T2A peptides [23]. Their approach resulted in eYFP expression in vivo with detectable signals for 24 h after footpad injection [23]. Our NovaVec used a different strategy, i.e., protein B2 coexpression design, to express foreign proteins by positioning the GOI ORF in sgRNA3 to coexpress with protein B2 that had minimal impact on protein A function. We pioneered the use of LNP to package the NoV RNA1-based saRNA vector, NovaVec, which enabled the Nluc expression in vivo for up to 54 days in mice. Delivery of NovaVec with LNP avoided potential limitations associated with pre-existing immunity to viral capsids, which may affect VLP-based approaches. NovaVec demonstrated a strong ability to sustain long-term protein expression over the LNP-mRNA approach and the previous protein A-coexpression-based VLP vector [23]. The protein A-coexpression-based VLP vector carrying the SARS-CoV-2 receptor binding domain protein can elicit an effective immune response in mice [25]. On the other hand, we developed an mpox vaccine using NovaVec in the current study, which induced a potent antigen-specific humoral immune response. Compared to the conventional LNP-mRNA approach and the previous NoV-based VLP vector, NovaVec stands out in sustaining foreign protein expression in vivo, ease of production and delivery, and potential applications. In this study, the evaluation of the immune response primarily focused on antibody levels as a preliminary validation of NovaVec’s efficacy as a vaccine vector. While cellular immunity is essential for a comprehensive vaccine evaluation, our previous research on the A33R antigen using an mRNA-LNP platform has already established its capacity to elicit both robust humoral and antigen-specific T-cell responses, including CD8+ T cells and Th1-biased CD4+ T cells [42]. Building on this established baseline, the robust IgG response observed here (Figure 5F,G) confirms the functional feasibility of NovaVec for vaccine delivery. Future studies utilizing the NovaVec vector for novel antigens will include comprehensive assessments of both humoral and cellular immune profiles to fully characterize their immunotherapeutic potential. Expanding studies of NovaVec and its optimization for further preclinical development would enrich the saRNA toolbox and unlock the promising value of NovaVec in medical therapeutics.

## 5. Conclusions

This study established NovaVec as a novel, compact and efficient saRNA platform, which was developed based on fundamental, detailed mechanism research and showed great promise for vaccine development. By elucidating the membrane-targeting determinants of NoV protein A in *S. pombe* and demonstrating the functional plasticity of its replication machinery, we provided a deeper biological understanding of the NoV replication mechanism and developed a practical saRNA platform tool for next-generation RNA vaccines.

## 6. Patents

P.H., X.L., X.S., and W.Q. are listed as inventors on Chinese patent application 2025109247188, entitled “A *Nodamura virus* (NoV)-based self-amplifying RNA vector”.

## Data Availability

The primer and expression plasmids used in this study are listed in Supplementary Tables. Any other data are included in the article and/or Supplementary Data.

## Supplementary Materials

The following supporting information can be downloaded at https://www.mdpi.com/article/10.3390/vaccines14060532/s1, Figure S1: Construction of Red-Mito yeast strain; Figure S2: Transmembrane domain prediction for FHV protein A and NoV protein A; Figure S3: Construction of Red-ER yeast strain; Figure S4: Integrating cDNA into yeast chromosomes induces more uniform and stable expression; Table S1: Co-localization Quantitative Analysis; Table S2: primer sequence; Table S3: Plasmids.
