# Supplementary material for: Deciphering the Nodamura virus Protein A Function in Schizosaccharomyces pombe and Engineering a Novel Self-Amplifying RNA (saRNA) Vector NovaVec for Vaccine Development
Source: Vaccines (Basel). 2026 Jun 15;14(6):532. doi: 10.3390/vaccines14060532 (PMC13308171; doi:10.3390/vaccines14060532)
Supplement: Supplementary file 1 [file vaccines-14-00532-s001.zip › vaccines-4315951-supplementary.pdf]

Supplementary Information

Deciphering Nodamura Virus protein A function in Schizosac-charomyces pombe and engineering a novel self-amplifying RNA (saRNA) vector NovaVec for vaccine development

Xueyao Song[1,4,#], Ruihan Liu[1,4,#], Zhuo Zhang[2,4], Yuying Pan[3],

Wanting Qu[1,4], Niubing Zhang[1], Xuan Li[1,4,*], Xiangping Yao[3,*], Pei Hao[2,4,*]

[1]Key Laboratory of Synthetic Biology, State Key Laboratory of Plant Trait Design, CAS Center for Excellence in Molecular Plant Sciences, Chinese Academy of Sciences, Shanghai 200032, China

[2]Shanghai Institute of Materia Medica, Chinese Academy of Sciences, Shanghai 13 201203, China

[3]Department of Neurology, Fujian Institute of Neurology, the First Affiliated Hospital, Fujian Medical University, Fuzhou, 350005, China

[4]University of Chinese Academy of Sciences, Beijing 100039, China

* Corresponding author

Email: lixuan@sippe.ac.cn (Xuan Li); yaoxiangping@fjmu.edu.cn (Xiangping Yao); phao@simm.ac.cn (Pei Hao)

A.

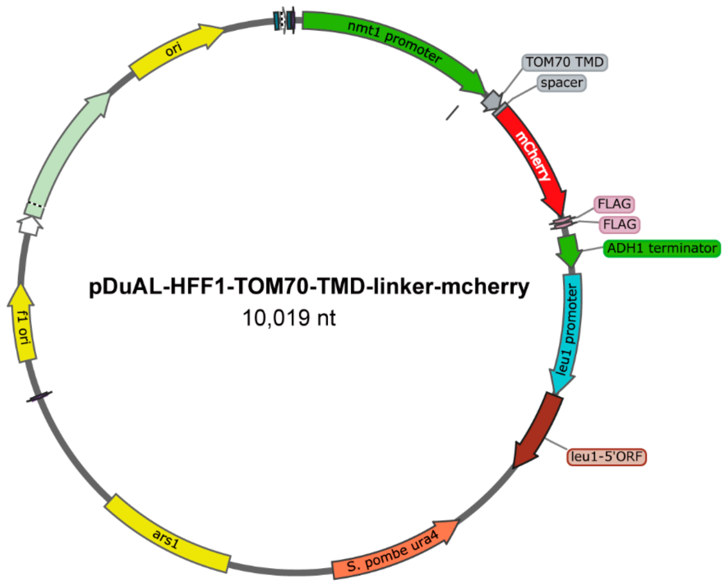

B.

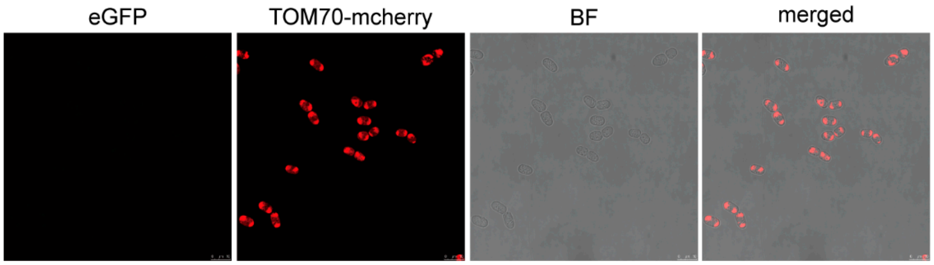

Supplementary Fig. S1.

Construction of Red-Mito yeast strain. (**A**) Plasmid map of pDuAL-HFF1-TOM70-TMD-linker-mCherry. (**B**) Representative fluorescent photographs of FY7652 cells with TOM70-linker-mCherry cDNA integrated into chromosomes.

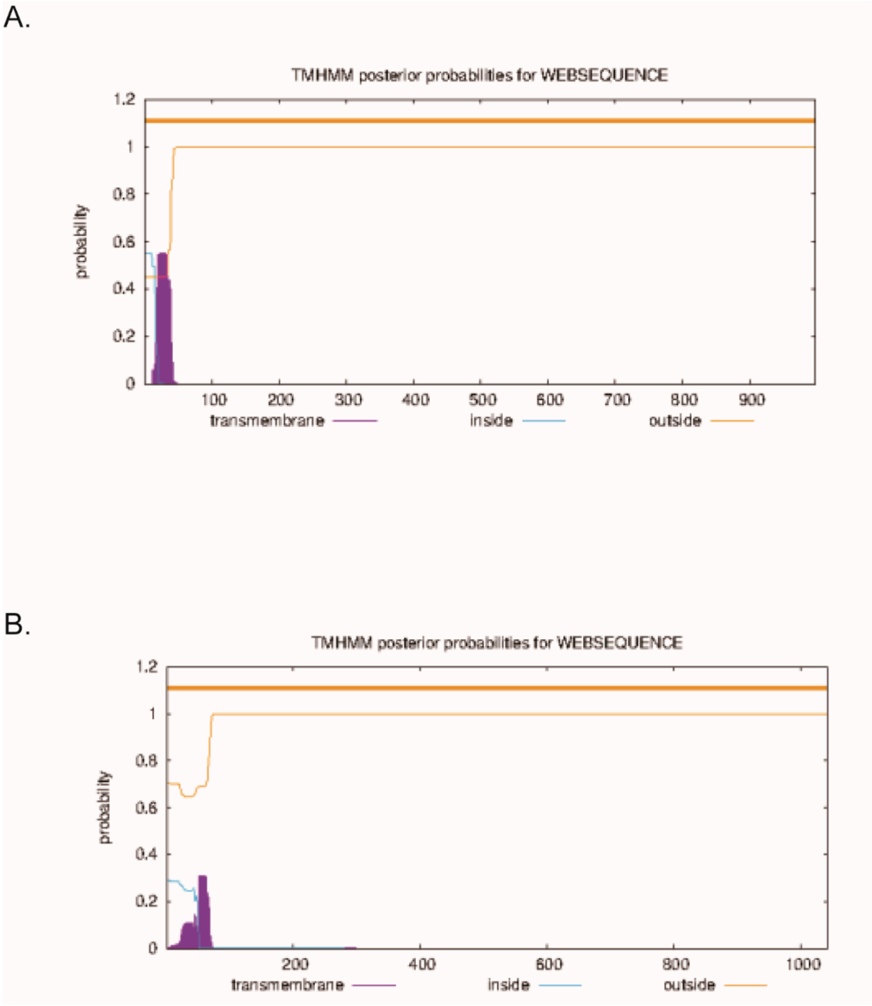

Supplementary Fig. S2.

Transmembrane domain prediction for FHV protein A and NoV protein A. (**A**) Transmembrane domain prediction for FHV protein A. (**B**) Transmembrane domain prediction for NoV protein A.

A.

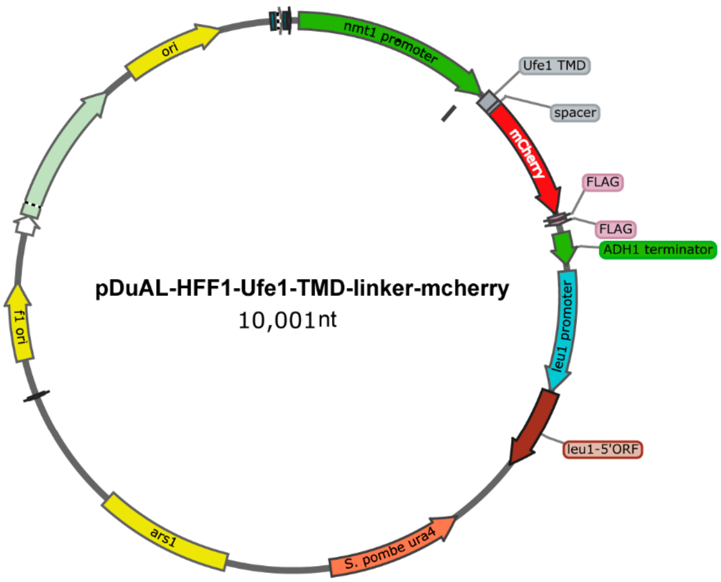

B.

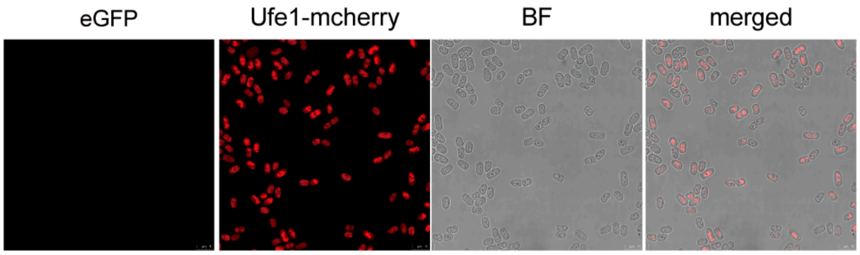

Supplementary Fig. S3.

Construction of Red-ER yeast strain. (**A**) Plasmid map of pDuAL-HFF1-Ufe1-TMD-linker-mCherry. (**B**) Representative fluorescent photographs of FY7652 cells with Ufe1-linker-mCherry cDNA integrated into chromosomes.

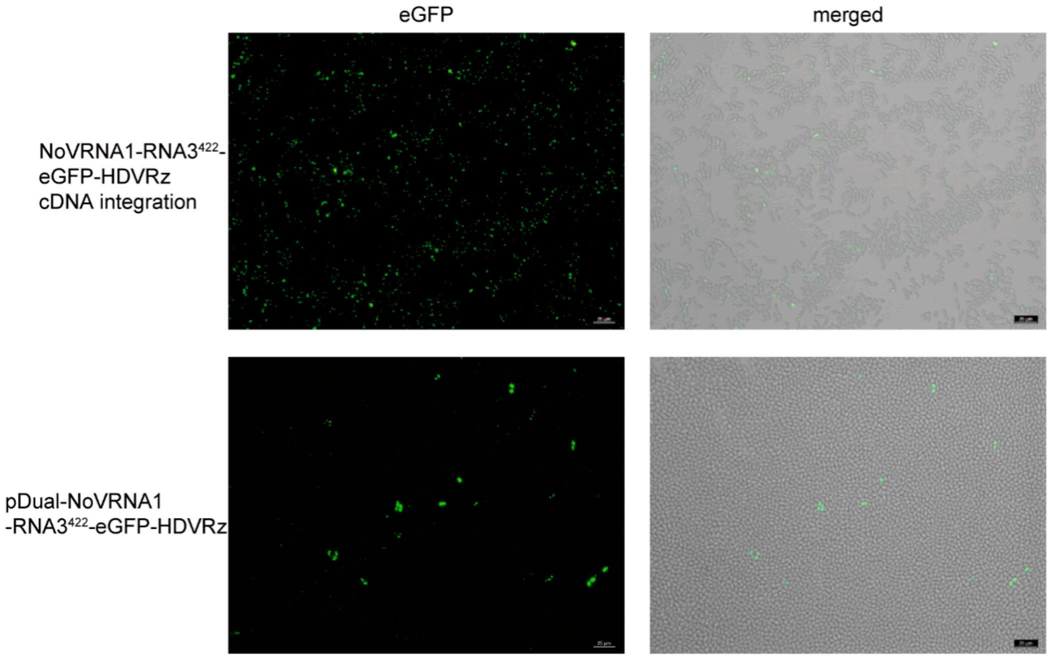

Supplementary Fig. S4.

Integrating cDNA into yeast chromosomes induces more uniform and stable expression. Representative fluorescence images of FY7652 cells. The first row of images represents the FY7652 cells integrated with NoVRNA1-RNA3^{422}-eGFP-HDVRz cDNA into chromosome; the second row of images represents the FY7652 cells transformed with pDuAL-NoVRNA1-RNA3^{422}-eGFP-HDVRz.

Supplementary Table S1. Co-localization Quantitative Analysis

Sample	PCC
Wt protein A with mitochondria	0.83
Δ2-15 protein A with mitochondria	0.89
Δ16-33 protein A with mitochondria	0.49
Δ2-33 protein A with mitochondria	＜0
Δ2-49 protein A with mitochondria	0.31
Ufe1-TMD protein A with ER	0.57

Notes: PCC, Pearson correlation coefficient is the most commonly used quantitative measure of colocalization correlation, with values ranging from 1 to -1. A value of 1 indicates perfect correlation; a value of -1 indicates complete exclusion.

Supplementary Table S2. primer sequence		
Primer name	Sequence (5′-3′)	Characteristics
nmt1-NoV-5UTR-RNA1-F1	TATAGTCGCTTTGTTAAAGCTAGCGTCGACGTATTGAATCCAAAACTCAAAATGCTGAACTAC	To clone pDuAL-protein A-eGFP
pDuAL-NdeI-NoVRNA1-R1	GTCATCATCCTTATAATCCATATGCGAGCTCTCCCTTAGCCATCCG	To clone pDuAL-protein A-eGFP
eGFP--NoVRNA1-F1	atggacgagctctacaagTGAGTGATTCATCGTCCCATCTGACG	To clone pDuAL-protein A-eGFP
eGFP-RNA1^{3151}-NoVRNA1-R1	ctcgcccttgctcaccatTTTACCACGCCCACGCGACCC	To clone pDuAL-protein A-eGFP
NoVRNA1-RNA1^{3151}-eGFP-F1	TCGCGTGGGCGTGGTAAAatggtgagcaagggcgagg	To clone pDuAL-protein A-eGFP
NoVRNA1-RNA1^{3151}-eGFP-R1	GGGACGATGAATCACTCActtgtagagctcgtccatgccg	To clone pDuAL-protein A-eGFP
NoVptnA-RNA3^{422}-eGFP-F1	GTGGGCGTGGTAAATGAGatggtgagcaagggcgag	To clone pDuAL-NoVRNA1-RNA3^{422}-eGFP-HDVRz
NoVRNA1-eGFP-R1	AGATGGGACGATGAATCActtgtagagctcgtccatgccg	To clone pDuAL-NoVRNA1-RNA3^{422}-eGFP-HDVRz
eGFP-RNA3^{422}-NoVptnA-R1	ctcgcccttgctcaccatCTCATTTACCACGCCCACGC	To clone pDuAL-NoVRNA1-RNA3^{422}-eGFP-HDVRz

eGFP-NoVRNA1-F1	gacgagctctacaagTGATTCATCGTCCCATCTGACGAAACC	To clone pDuAL-NoVRNA1-RNA3^{422}-eGFP-HDVRz
nmt1-SalI-TOM70-TMD-F1	TATAGTCGCTTTGTTAAAGCTAGCGTCGAATGCCAAAATCTTTTATTACTAGAAATAAAACTGCTATTTTGGCTGCTGTTGCTGCTACTGGTAC	To clone pDuAL-TOM70-linker-mCherry
mCherry-linker-TOM70-TMD-R1	ctcgcccttgctcaccatACCACCAGAACCACCAGAACCACCATAATAATAATAAGCACCAATAGCAGTAGTACCAGTAGCAGCAACAGCAG	To clone pDuAL-TOM70-linker-mCherry
liner-mCherry-F1	TCTGGTGGTTCTGGTGGTatggtgagcaagggcgagg	To clone pDuAL-TOM70-linker-mCherry
pDuAL-mCherry-R1	CATCATCCTTATAATCCATATGctacttgtacagctcgtccatgc	To clone pDuAL-TOM70-linker-mCherry
nmt1-SalI-Ufe1-f1	TATAGTCGCTTTGTTAAAGCTAGCGTCGAatgAGAACTAAAGGTGTTTATGATTTGTTTTTGATTTTTG	To clone pDuAL-Ufe1-linker-mCherry
mCherry-linker-Ufe1-R1	ctcgcccttgctcaccatACCACCAGAACCACCAGAACCACCTTTCAAAGTAGTATAACCAGCAATAATCATACCAAC	To clone pDuAL-Ufe1-linker-mCherry
nmt1-5UTR-del2-15-F1	CGCTTTGTTAAAGCTAGCGTCGACGTATTGAATCCAAAACTCAAAATGAACATCGTTTCGCGTGCG	To clone pDuAL-NoVRNA1-△2-15-RNA3^{422}-eGFP-HDV Rz
lin-nmt1-R2	cTTTAACAAAGCGACTATAAGTCAGAAAGTGAG	To clone pDuAL-NoVRNA1-△2-15-RNA3^{422}-eGFP-HDVRz

ADH-term-F	CTCTTATTGACCACACCTCTACC	To clone pDuAL-NoVRNA1-△2-15-RNA3^{422}-eGFP-HDVRz
RSR1	CAATATGGTCTCCTGGTAAGACGACG	To clone pDuAL-NoVRNA1-△2-15-RNA3^{422}-eGFP-HDVRz
nmt1-5UTR-del2-33-F1	CGCTTTGTTAAAGCTAGCGTCGACGTATTGAATCCAAAACTCAAAATGCTGGTCGCGGGGTCCTG	To clone pDuAL-NoVRNA1-△2-33-RNA3^{422}-eGFP-HDVRz
NoV-SR1	CCAGGAACGCCACGAGC	To clone pDuAL-NoVRNA1-△2-33-RNA3^{422}-eGFP-HDVRz
NoV-SF2	CGATTACTACCTGCGTGACATTG	To clone pDuAL-NoVRNA1-△2-33-RNA3^{422}-eGFP-HDVRz
nmt1-5UTR-del2-49-F1	CGCTTTGTTAAAGCTAGCGTCGACGTATTGAATCCAAAACTCAAAATGACGCTCGTGGCGTTCC	To clone pDuAL-NoVRNA1-△2-49-RNA3^{422}-eGFP-HDVRz
4bp-T7-AGG-2-49-F1	tactTAATACGACTCACTATAAGGGTATTGAATCCAAAACTCAAAATGACGCTCG	To clone pDuAL-NoVRNA1-△2-49-RNA3^{422}-eGFP-HDVRz
nmt1-5UTR-NoV-delmb-F1	TATAGTCGCTTTGTTAAAGCTAGCGTCGACGTATTGAATCCAAAACTCAAAATGCTGAACTACGAGACAATCATCAACGGCGCATCGAGCGCTCTGCTGGTCGCGGGGTCCT	To clone pDuAL-NoVRNA1-△16-33-RNA3^{422}-eGFP-HDVRz

NoVptnA-deletion1-F1	GGCGCATCGAGCGCTCTGCTGGTCGCGGGGTCCTG	To clone pDuAL-NoVRNA1-△16-33-RNA3^{422}-eGFP-HDVRz
NoVptnA-deletion1-R1	GCAGGACCCCGCGACCAGCAGAGCGCTCGATGCGC	To clone pDuAL-NoVRNA1-△16-33-RNA3^{422}-eGFP-HDVRz
NoV-SF3	GACATGCTTATGGGTTTGTCGAG	To clone pDuAL-NoVRNA1-△16-33-RNA3^{422}-eGFP-HDVRz
NoV-SF4	CCGACCATGAAGGCTGG	To clone pDuAL-NoVRNA1-△16-33-RNA3^{422}-eGFP-HDVRz
Ufe1TMD-F1	AGAACTAAAGGTGTTTATGATTTGTTTTTGATTTTTGTTG	To clone pDuAL-NoVRNA1-△16-33-RNA3^{422}-eGFP-HDVRz
delmb-Ufe1TMD-R1	ATAAACACCTTTAGTTCTCAGAGCGCTCGATGCG	To clone pDuAL-NoVRNA1-△16-33-RNA3^{422}-eGFP-HDVRz
NoV1-15-Ufe1TMD-F1	GGCGCATCGAGCGCTCTGAGAACTAAAGGTGTTTATGATTTGTTTTTGATTTTTGTTG	To clone pDuAL-NoVRNA1-△16-33-RNA3^{422}-eGFP-HDVRz
nmt1-5UTR-yeast Ufe1-NoVRNA1-F1	TATAGTCGCTTTGTTAAAGCTAGCGTCGACGTATTGAATCCAAAACTCAAAatgAGAACTAAAGGTGTTTATGATTTGTTTTTGATTTTTGTTGGTATGATTATTGCTGGTTATACTACTTTGAAACTGGTCGCGGGGTCCTG	To clone pDuAL-NoVRNA1-RNA3^{422}-eGFP-HDVRz (Ufe1-TMD)

SSF1	CTCACTTTCTGACTTATAGTCGCTTTG	To clone pDuAL-NoVRNA1-RNA3^{422}-eGFP-HDVRz (Ufe1-TMD)
RZ-SF1	ggcatggcatctccacctcc	To clone pDuAL-NoVRNA1-RNA3^{422}-eGFP-HDVRz (Ufe1-TMD)

Supplementary Table S3. Plasmids

Plasmids	Characteristics	Source
pMT-NoVRNA1	NOV RNA1 (accession: AF174533)	kindly provided by Dr. Li's Lab, FDU
pDuAL-HFF1	AmpR，S. pombe expression vector，nmt1 promoter	Laboratory Storage
pCDNA3.1(+)	AmpR，Mammalian Expression Vectors，SV40 promoter	Laboratory Storage
pGEX-4T-1	AmpR，Bacterial vector for expressing GST-tagged fusion proteins with a thrombin site.	Laboratory Storage
pCDNA3.1-ScaI-T7-NoVRNA1-RNA3^{422}-T2A-A33R-3'RE-globin-117A	AmpR，RNA3^{422}-NoVRNA1，A33R	The author constructs
pCDNA3.1-ScaI-T7-NoVRNA1-RNA3^{422}-T2A-Nluc-3'RE-globin-117A	AmpR，RNA3^{422}-NoVRNA1，Nluc	The author constructs
pDuAL-TOM70-TMD-linker-mCherry	AmpR，TOM70-TMD，mCherry	The author constructs
pDuAL-Ufe1TMD-linker-mCherry	AmpR，Ufe1TMD，mCherry	The author constructs
pDuAL-HFF1-NoVRNA1-RNA1^{3151}-eGFP-HDVRz	AmpR，NoVRNA1-RNA1^{3151}-eGFP	The author constructs
pDuAL-HFF1-NoVRNA1-RNA3^{422}-eGFP-HDVRz	AmpR，NoVRNA1-RNA3^{422}-eGFP	The author constructs

pDuAL-HFF1-NoVRNA1-deletion2-15-RNA1^{3151}-eGFP-HDVRz	AmpR，△2-15-RNA1^{3151}，eGFP	The author constructs
pDuAL-HFF1-NoVRNA1-deletion2-15-RNA3^{422}-eGFP-HDVRz	AmpR，△2-15-RNA3^{422}，eGFP	The author constructs
pDuAL-HFF1-NoVRNA1-deletion2-33-RNA1^{3151}-eGFP-HDVRz	AmpR，△2-33-RNA1^{3151}，eGFP	The author constructs
pDuAL-HFF1-NoVRNA1-deletion2-33-RNA3^{422}-eGFP-HDVRz	AmpR，△2-33-RNA3^{422}，eGFP	The author constructs
pDuAL-HFF1-NoVRNA1-deletion2-49-RNA1^{3151}-eGFP-HDVRz	AmpR，△2-49-RNA1^{3151}，eGFP	The author constructs
pDuAL-HFF1-NoVRNA1-deletion2-49-RNA3^{422}-eGFP-HDVRz	AmpR，△2-49-RNA3^{422}，eGFP	The author constructs
pDuAL-HFF1-NoVRNA1-deletion2-49-Ufe1-RNA3^{422}-eGFP-HDVRz	AmpR，△2-49-Ufe1-RNA3^{422}，eGFP	The author constructs
pDuAL-HFF1-NoVRNA1-deletion16-33-RNA1^{3151}-eGFP-HDVRz	AmpR，△16-33-RNA1^{3151}，eGFP	The author constructs
pDuAL-HFF1-NoVRNA1-deletion16-33-RNA3^{422}-eGFP-HDVRz	AmpR，△16-33-RNA3^{422}，eGFP	The author constructs
pDuAL-HFF1-NoVRNA1-yeast Ufe1-RNA1^{3151}-eGFP-HDVRz	AmpR，yeast Ufe1-RNA1^{3151}，eGFP	The author constructs
pDuAL-HFF1-NoVRNA1-yeast Ufe1-RNA3^{422}-eGFP-HDVRz	AmpR，yeast Ufe1-RNA3^{422}，eGFP	The author constructs
pGEX-4T-1-A33R	MPV-antigen A33R	The author constructs
